# Supplementary material for: Astrocyte reactivity and inflammation-induced depression-like behaviors are regulated by Orai1 calcium channels
Source: Nat Commun. 2023 Sep 7;14:5500. doi: 10.1038/s41467-023-40968-6 (PMC10485021; doi:10.1038/s41467-023-40968-6)
Supplement: Supplementary file 1 — Supplementary Information [file 41467_2023_40968_MOESM1_ESM.pdf]

Supplementary Material

Astrocyte reactivity and inflammation-induced depression-like behaviors are regulated by Orai1 calcium channels

Michaela M. Novakovic[1], Krill S. Korshunov[1], Rogan A. Grant[2], Hiam A. Valencia[2], Megan M Martin[1], GR Scott Budinger[2], Jelena Radulovic[3], and Murali Prakriya[1]

[1]Department of Pharmacology, Northwestern University Feinberg School of Medicine, Chicago, IL 60611, USA, [2]Department of Medicine, Northwestern University Feinberg School of Medicine, Chicago, IL 60611, USA [3]Department of Neuroscience, Albert Einstein School of Medicine, Bronx, NY 10461, USA.

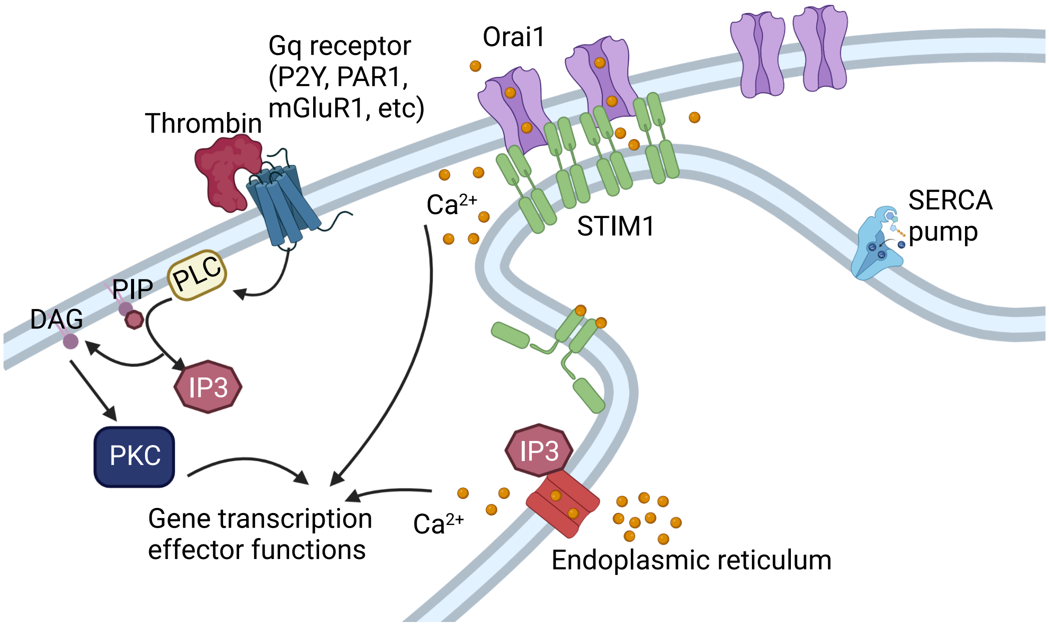

Supplementary Figure 1: Scheme illustrating store-operated calcium entry (SOCE) and Orai1 Ca^{2+} signaling in astrocytes. G-protein coupled receptor activation by thrombin or ATP results in the cleavage of PIP_2 to diacyl glycerol (DAG) and inositol triphosphate (IP3), causing activation of protein kinase C (PKC), and depletion of endoplasmic reticulum (ER) ER Ca^{2+} stores. The ER Ca^{2+} sensor, STIM1, senses depletion of ER Ca^{2+} stores and activates Orai1 via direct binding to trigger SOCE. Thapsigargin (TG) blocks the SERCA pump to deplete ER Ca^{2+} stores directly and activate SOCE. Illustration created with BioRender.com.

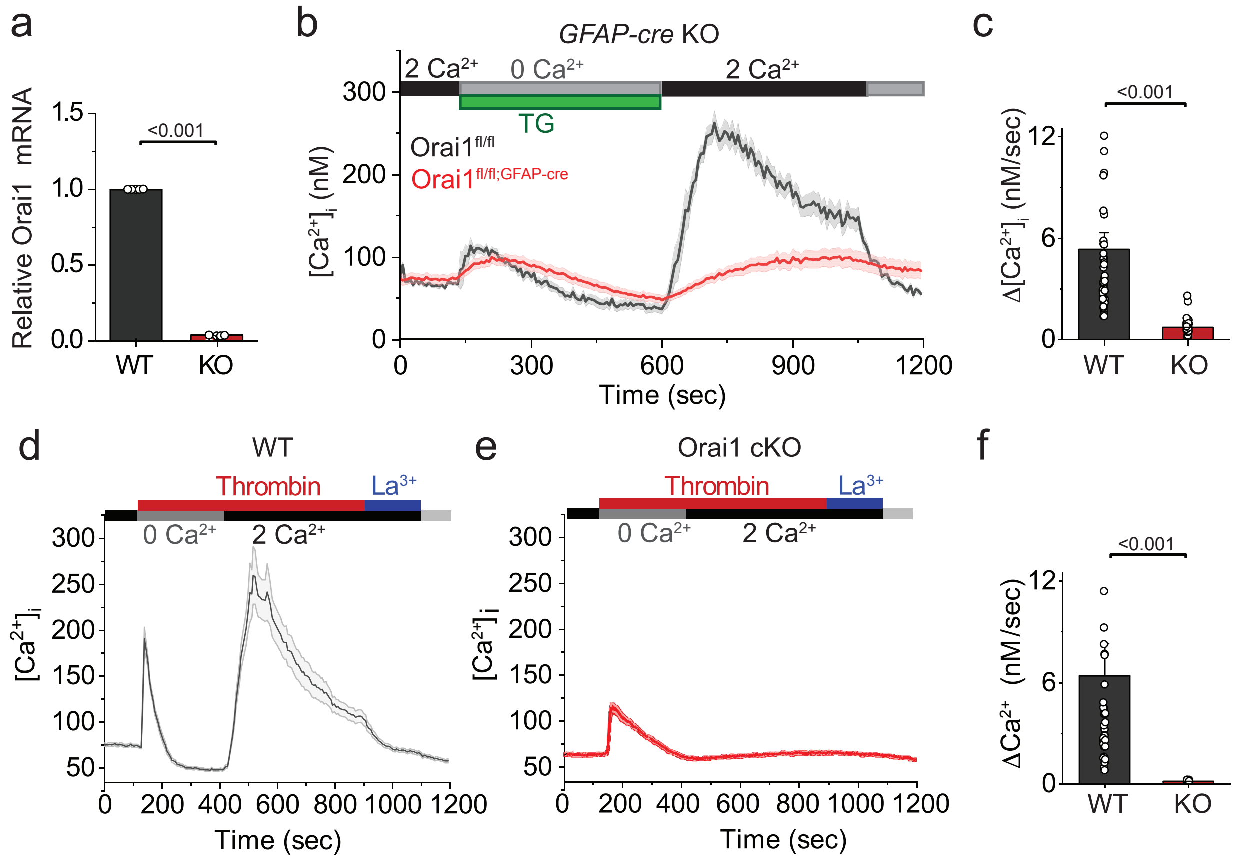

Supplementary Figure 2: Orai1 mediates SOCE in hippocampal astrocytes. (a) Orai1$^{fl/fl\ GFAP\ Cre}$ astrocytes show loss of Orai1 mRNA relative to WT (Orai1$^{fl/fl}$) controls. (**B,C)** SOCE was induced by depleting ER Ca^{2+} stores with thapsigargin (TG, 1 µM) applied in a 0 mM Ca^{2+} Ringer's solution. Re-addition of extracellular Ca^{2+} (2 mM) reveals SOCE in WT but not in Orai1$^{fl/fl\ GFAP\text{-}Cre}$ astrocytes. Panel *C* summarizes the rate of Ca^{2+} influx (nM/sec) following re-addition of extracellular Ca^{2+} following store depletion. **(d-f)** Thrombin (1 U/mL) induces store release and SOCE in WT *(d)* but not Orai1 KO *(e)* astrocytes. SOCE rates for thrombin are summarized in panel *f*. Data are presented as mean values +/- SEM. *(a)* n=5 mice/group; *(c)* n=60 cells from 4 WT mice; n=50 cells from 3 KO mice; and *(f)* n=36 cells from 3 WT mice; n=27 cells from 3 KO mice, sex was not determined. Statistical tests were conducted by two-tailed, unpaired T-tests. Source data are provided as a Source Data file.

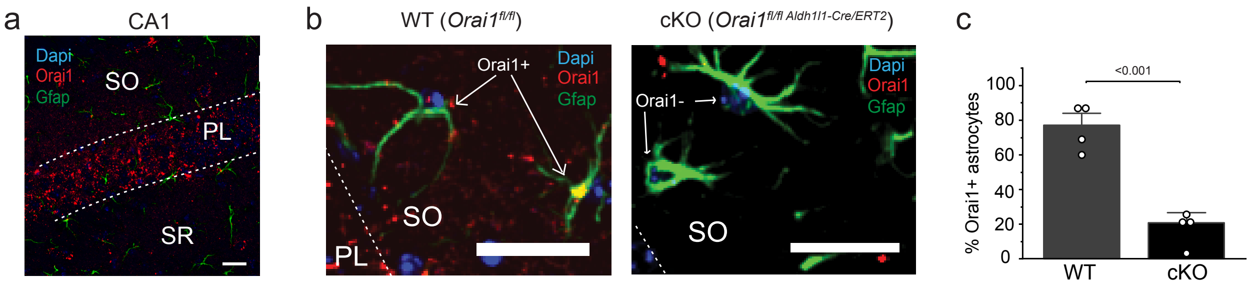

Supplementary Figure 3: Immunohistochemistry of Orai1 in the murine hippocampus. (a) Orai1 (depicted in red) is expressed in the CA1 region of the hippocampus, particularly in the pyramidal cell layer (PL), but labelling is also seen in the *Stratum Oriens (SO)* and *Stratum Radiatum (SR)* regions. Sections are co-labeled with GFAP (shown in green) to identify astrocytes. Images are representative of data from 4 WT mice. **(b)** Higher magnification image showing astrocytes in the *SO* region co-labeled for Orai1 in WT mice. Orai1 cKO (Orai1$^{fl/fl\ Aldh1l1\text{-}Cre/ERT2}$) mice show far less Orai1-GFAP colocalization than that seen in WT mice. **(c)** Quantification of cells co-labeled with GFAP and Orai1. Scale bars in all panels are 25 μm. Number of mice: WT: n=3 male, 1 female; Orai1 cKO: n= 2 male, 2 female. Data are presented as mean values +/- SEM**.** Statistical tests were conducted by two-tailed, unpaired T-test ($p=8.95x10^{-4}$). Source data are provided as a Source data file.

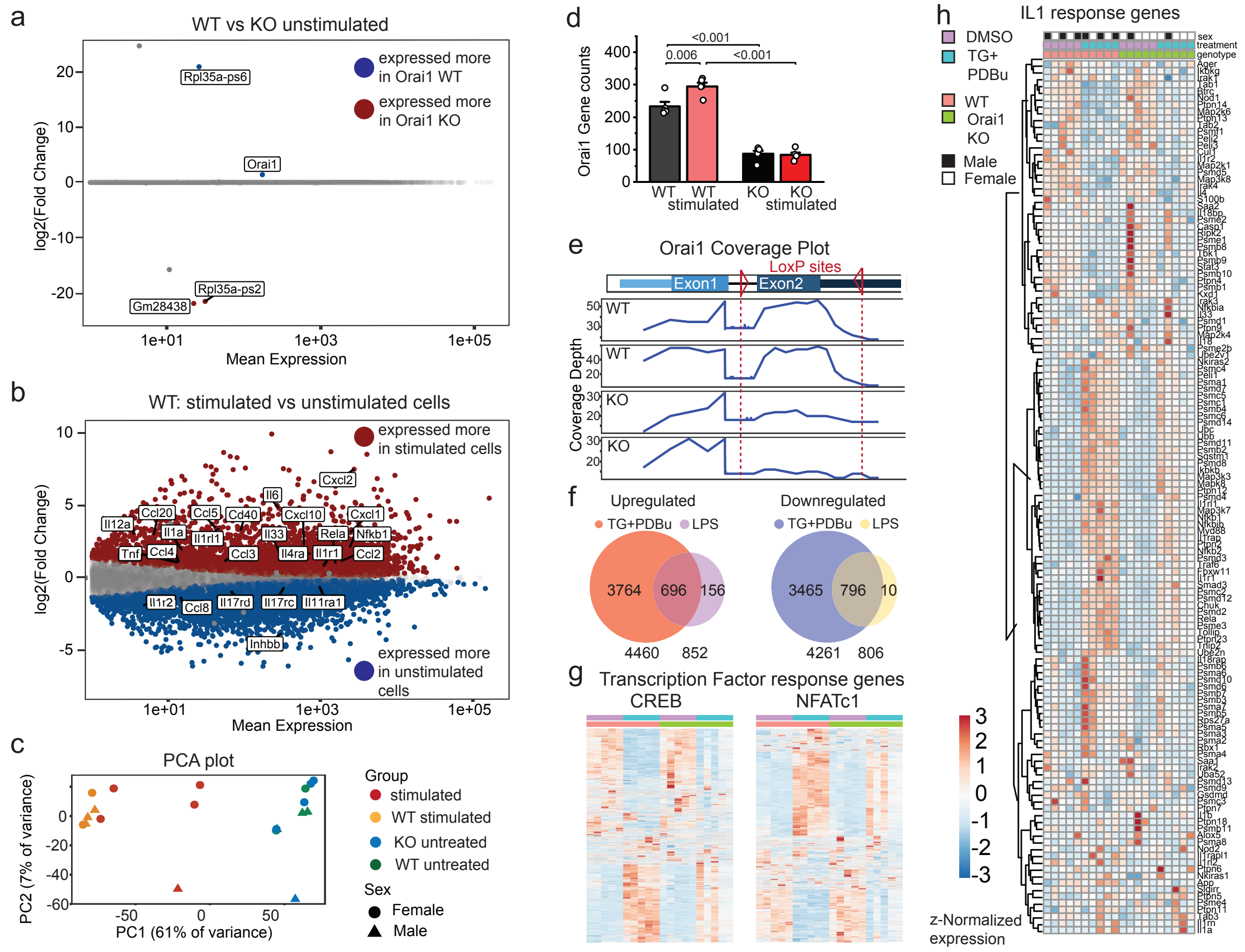

Supplementary Figure 4: Stimulation of SOCE drives large-scale transcriptional changes in hippocampal astrocytes. RNA-sequencing analysis of Orai1$^{fl/fl}$ (WT) and Orai1$^{fl/fl\ GFAP\text{-}Cre}$ (cKO) astrocytes stimulated with 0.2 µM TG + 50 nM PDBu (or DMSO) for 6 hours. **(a)** In resting cells, only Orai1 and three other genes of unknown function, Rpl35a-ps2, Gm28438, Rpl35a-ps6, are altered in Orai1 KO astrocytes. **(b)** By contrast, 10,840 mRNAs are significantly different between WT astrocytes after cell stimulation with TG+PDBu and resting WT cells. Gray dots indicate genes not significantly different between conditions while red and blue dots represent genes that were expressed differently. Statistics were performed using the Wald test, alpha is $p<0.05$, adjusted for multiple comparisons. **(c)** PCA analysis. The primary variance was driven by treatment, with additional contribution of genotype after treatment. **(d)** Orai1 gene counts. cKO cells show marked decrease in Orai1 mRNA. (WT: n=3 male, 2 female mice; KO: n=1 male, 4 female mice). Data are presented as mean +/- SEM. Statistical tests were conducted by two-way ANOVA followed by Tukey posthoc test. **(e)** Orai1 gene coverage analysis in WT and cKO cells. In the cKO cells, the detected Orai1 transcript is localized primarily to regions outside the coding mRNA template not excised by the Cre/LoxP system. The coding region for Orai1 is entirely in exon 2 and is excised by Cre. **(f)** Venn diagrams illustrating overlap in gene expression changes after TG+PDBu *in vitro* and LPS-injection *in vivo* (from ref. 30). **(g)** Heatmaps of z-normalized within-sample expression of significantly expressed CREB and NFATc1 response genes (HOMER analysis and compiled by the ENCODE project). Both genotype and treatment contribute to gene expression regulated by these transcription factors. **(h)** Full complement of the IL-1 responsive genes in the different genotypes. Source data are provided as a Source data file.

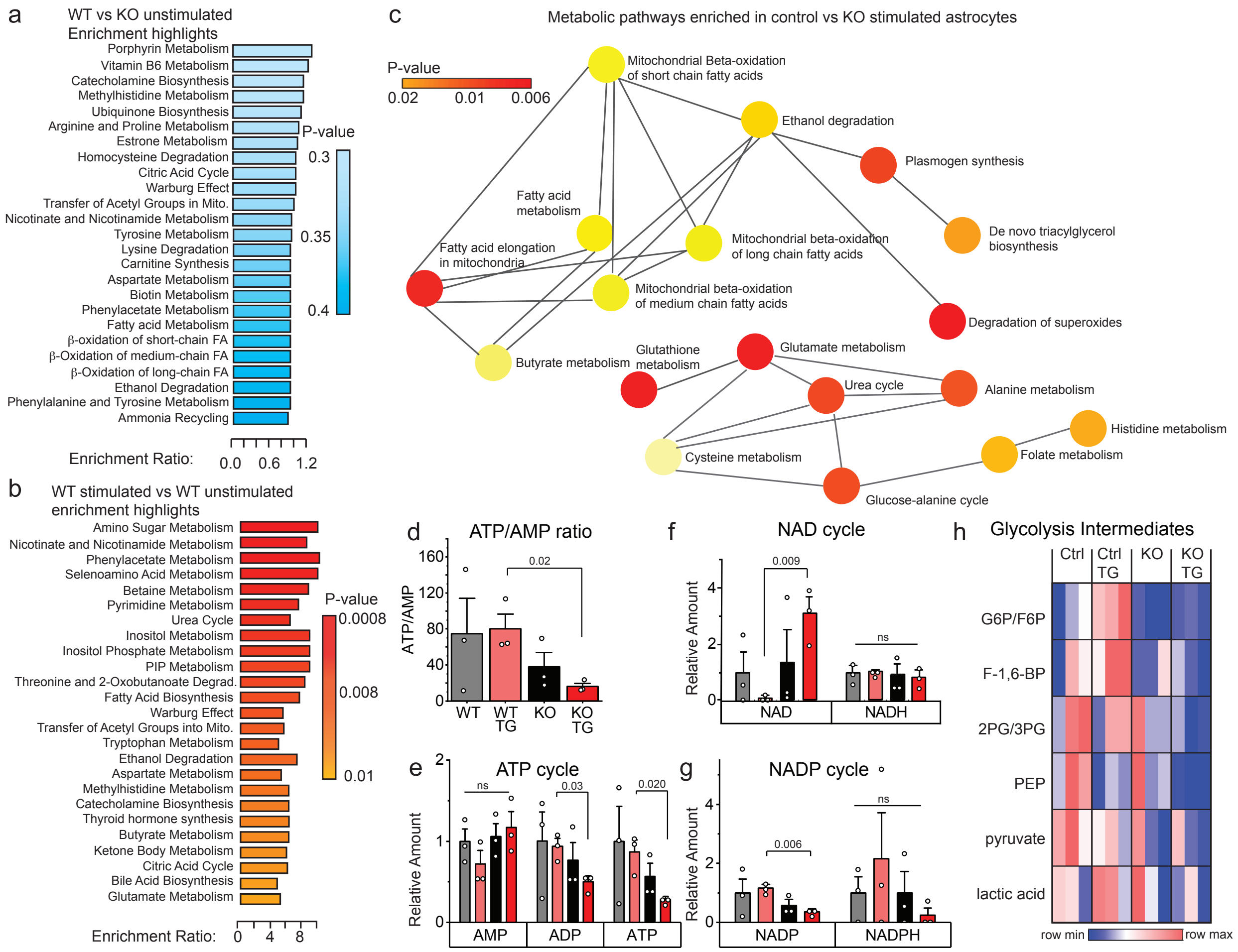

Supplementary Figure 5: Orai1 signaling drives metabolic activity in astrocytes. (a,b) Enrichment highlights of the 25 most enriched pathways found by Quantitative Enrichment Analysis (QEA) (metaboanalyst.ca). *(a)* No pathways are significantly altered in resting control astrocytes relative to Orai1 KO astrocytes. *(b)* Following cell stimulation with 0.2 µM TG + 50 nM PDBu (or DMSO control) for 6 hours, multiple metabolic pathways are altered in activated cells relative to resting cells. (p-values for Quantitative Enrichment Analysis were calculated using Global Test). **(c)** Pathway enrichment cluster plot showing relationships between different affected metabolic pathways. **(d-g)** Relative metabolite amounts in unstimulated and stimulated astrocytes. *(d)* ATP/AMP ratio. *(e)* ATP cycle, *(f)* NAD^+/NADH cycle, and *(g)* $NADP^+$/NADPH. No significant differences are seen in Orai1 cKO cells. Cell stimulation evokes opposite effects in control and KO cells in key metabolites: In control cells, the ATP/AMP ratio is slightly increased/maintained but NAD^+ is decreased. By contrast, cell stimulation decreases ATP/AMP ratio in the KO cells and increases NAD^+ levels. **(h)** Relative amounts of glycolysis intermediates. Data are shown as a heat map with red depicting the maximum and blue the minimum for each row. N=3 mice/group; sex was not determined. *d-g:* Data are presented as mean values +/- SEM. Statistical tests were conducted by two-tailed, unpaired T-tests. Source data are provided as a Source Data file.

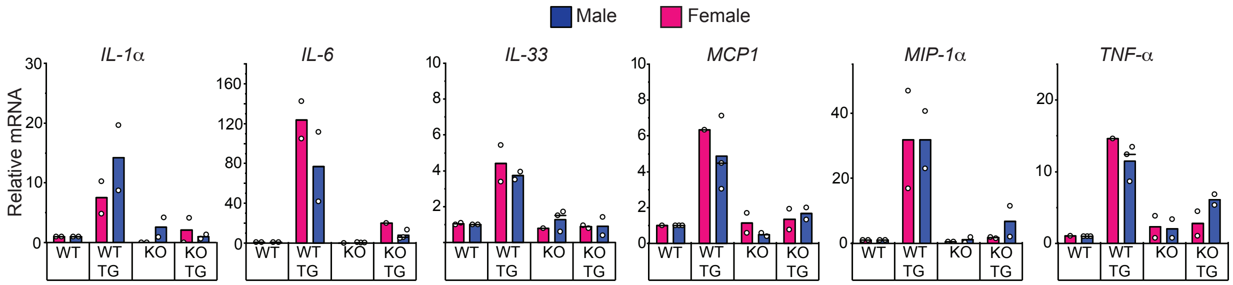

Supplementary Figure 6: Orai1 activation stimulates cytokine induction in mouse astrocytes in both sexes. Astrocyte were stimulated with TG+PDBu to induce pro-inflammatory cytokines (*IL-1α, IL-6, IL-33, MCP1, MIP-1α,* and *TNF-α*). When data are separated by sex, the trends of the cytokine levels and effect of ablating Orai1 are comparable between male and female mice. n=2 mice/group except TNF-α and MCP1 (WT: n=1 female, 3 male) and IL-6 (KO: 1 female, 3 male). No statistics as sample size is too small. Source data are provided as a Source Data file.

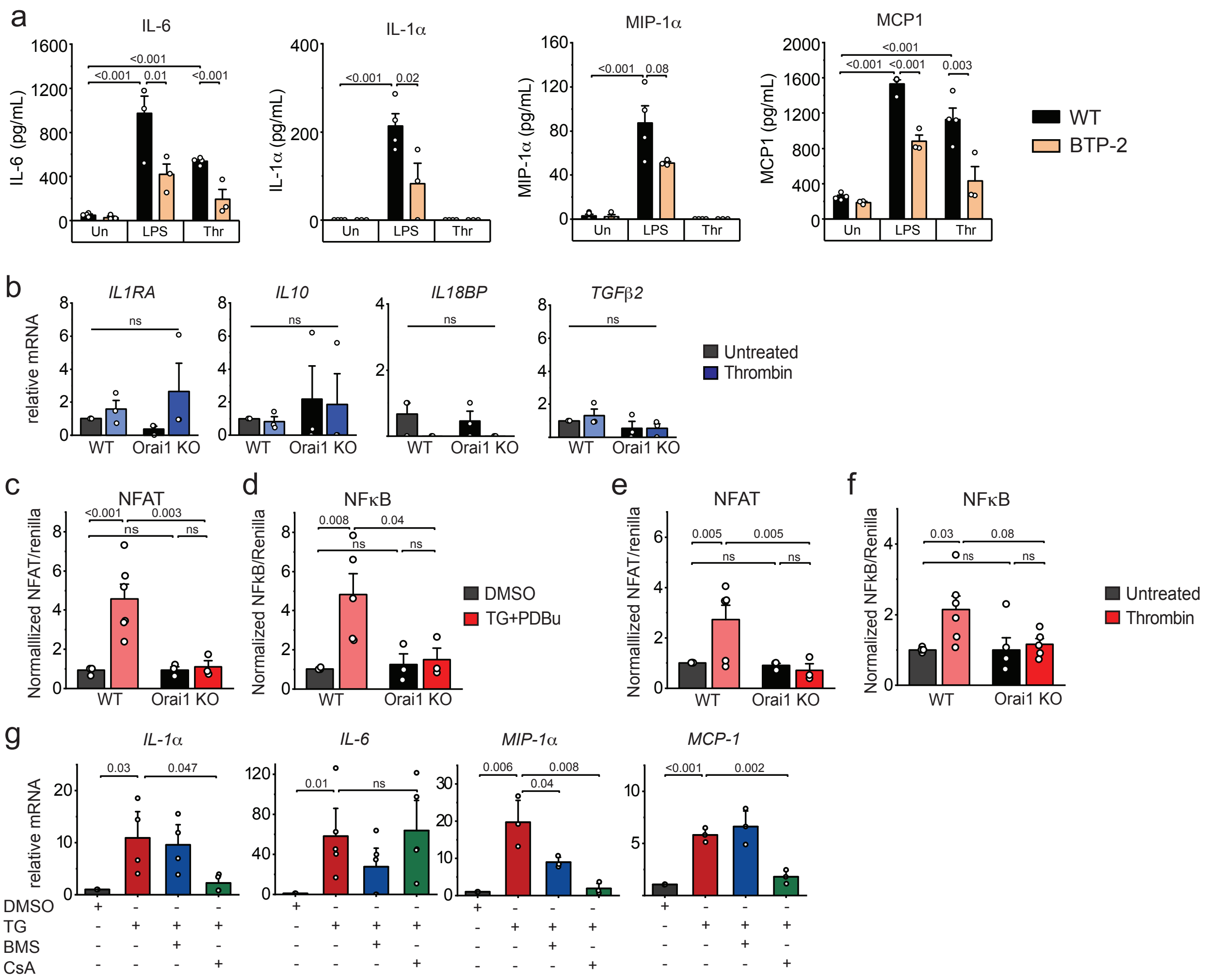

Supplementary Figure 7: Thrombin and LPS stimulate production of pro-inflammatory cytokines and NFAT and NFκB activation in an Orai1-dependent manner. (a) ELISAs measuring production of inflammatory cytokines. Cells were stimulated with LPS (100 nM) or thrombin (10 U/mL) for 24 hours. LPS increased all the cytokines examined, and induction of IL-6, IL-1α, and MCP1 was diminished in Orai1 WT astrocytes treated with 1 μM BTP-2. IL-1α and MIP-1α remained undetectable after thrombin stimulation, but IL-6 and MCP1 increased in an Orai1-dependent manner. (n=4 WT, 3 BTP-2 mice for each treatment). Two-way ANOVA with posthoc Tukey test was performed for each treatment independently. **(b)** Levels of the anti-inflammatory cytokines IL-10, IL-18 BP, TGFβ-2, and IL-1RA after 10 U/mL thrombin treatment measured by RT-qPCR. No difference is seen between groups (n=3 mice for group). **(c-f)** Activation of Orai1 stimulates NFAT and NFκB reporter expression. Transcription was assessed using NFAT::firefly or NFκB::firefly luciferase assays. TG+PDBu induces both NFAT *(c)* and NFκB *(d)* activity in WT but not Orai1 KO astrocytes. (*c*: n=6 WT, 3 KO mice, *d*: n=5 WT, 3 KO mice). Likewise, thrombin induces both NFAT *(e)* and NFκB *(f)* activity in WT but not Orai1 KO astrocytes (*e:* n=6 WT, 3 KO mice, *f:* n= 6 WT, 5 KO mice). Reporter activity was measured 4-hours after stimulation with TG (0.5 μM)+PDBu (50 nM) or Thrombin (2 U/mL). **(g)** Induction of *IL-1α* and *MCP1* is blocked by the NFAT/calcineurin inhibitor, cyclosporin (CsA, 25 nM) but not by the NFκB inhibitor BMS-35548 (BMS, 10 nM). By contrast, *MIP-α* induction is blocked by BMS and CsA, while *IL-6* induction is modestly blocked by BMS but not by CsA. (n=4 mice/group for *IL-6* and *IL-1α*, n=3 mice/group for *MIP-1α* and *MCP1*. Statistical tests were conducted by two-tailed unpaired T-tests. *(a-f)* Statistical tests were conducted by two-way ANOVA followed by Tukey posthoc tests. Sex was not determined. Source data are provided as a Source Data file.

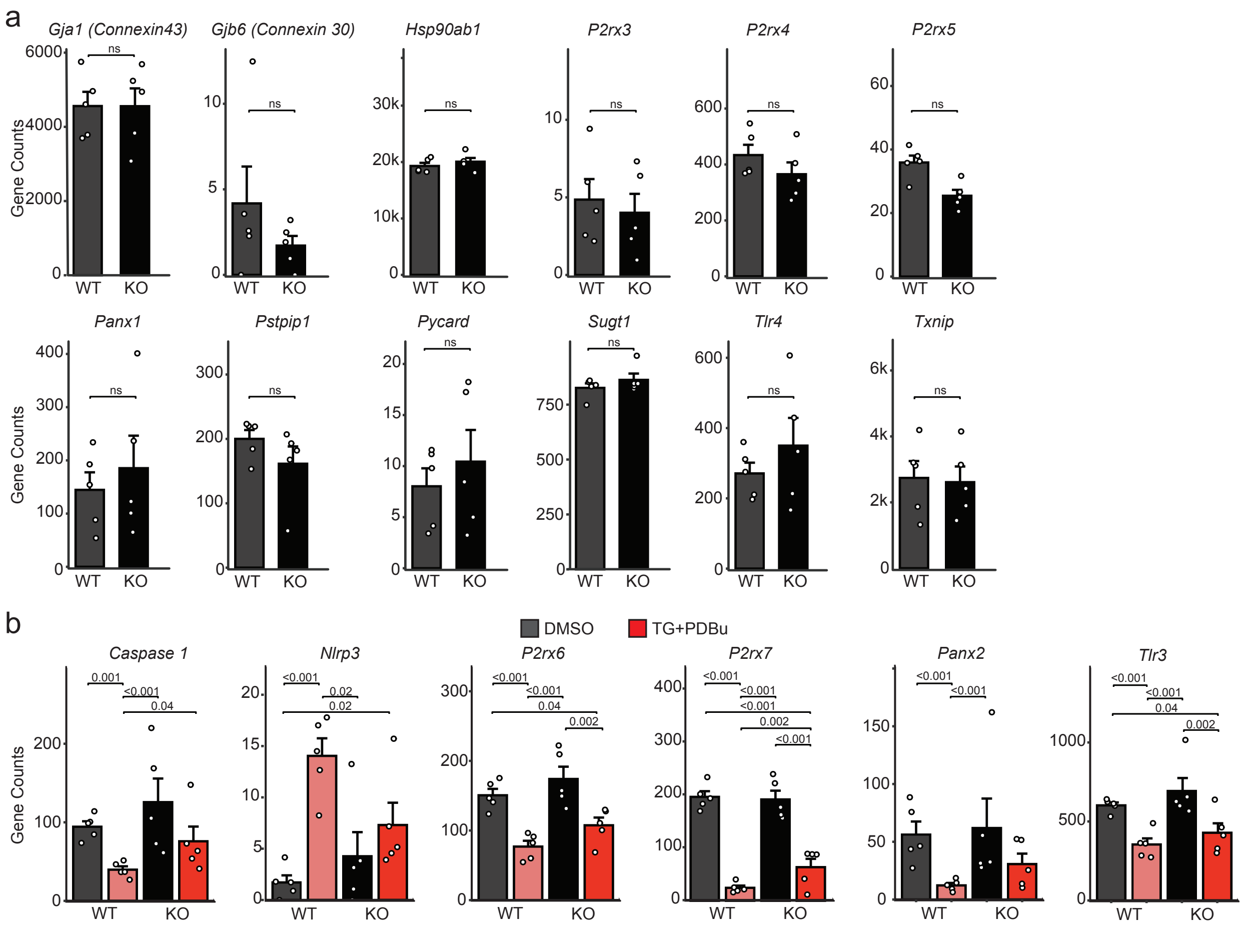

Supplementary Figure 8: RNA-Seq analysis of molecules involved in the LPS inflammatory response. (a) Normalized gene counts for genes known to be involved in LPS induced inflammatory responses and inflammasome pathway (TLRs, P2RXs, Connexins, Pannexins, etc.). Expression was not altered in Orai1 cKO astrocytes. **(b)** Stimulated cells show marked downregulation of several genes including P2RX7, TLR4, and Panx2. The extent of downregulation of these mRNA was similar in WT and Orai1 cKO astrocytes, indicating that while cell stimulation modulates expression of genes in the LPS/inflammasome pathways, this is not Orai1 dependent. For group-wise comparisons, statistical significance was calculated using Wald tests. (WT: n=3 male, 2 female mice; KO: 1 male, 4 female mice). Source data are provided as a Source Data file.

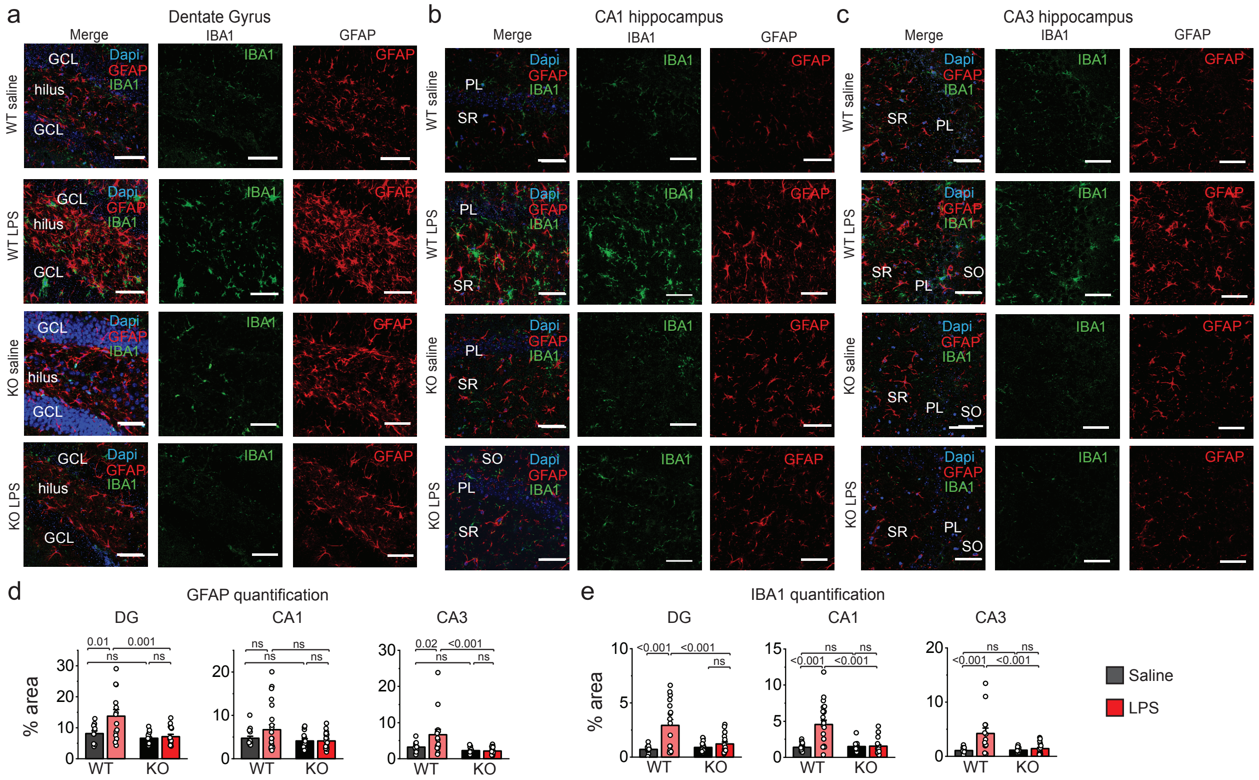

Supplementary Figure 9: Upregulation of astrocyte and microglial reactivity by LPS is blunted in Orai1*fl/fl Aldh1l1-Cre/ERT2* female mice. **(a-c)** Immunohistochemistry of hippocampal brain slices from Orai1*fl/fl* (WT) and Orai1*fl/fl Alh1l1-Cre/ERT2* (Orai1 cKO) female mice stained for GFAP and IBA1 antibodies. Mice were administered LPS or saline by intraperitoneal injection. Images show GFAP and IBA1 expression in the dentate Gyrus (DG, *A*), the CA1 region *(B)* and the CA3 region *(C)*. Scale bar = 50 µM. ***(d,e)*** Quantification of GFAP and IBA1 labeling in hippocampal slices from female mice. Data are given as means +/- SEM (WT saline: n=12-16 images from 3 mice; WT LPS: n=14-22 images from 5 mice, cKO saline: n=13-16 images from 3 mice; cKO LPS: n=15-20 images from 4 mice. All data are from female mice.) Statistical tests were conducted by two-way ANOVA followed by Tukey posthoc tests in each graph. Source data are provided as a Source Data file.

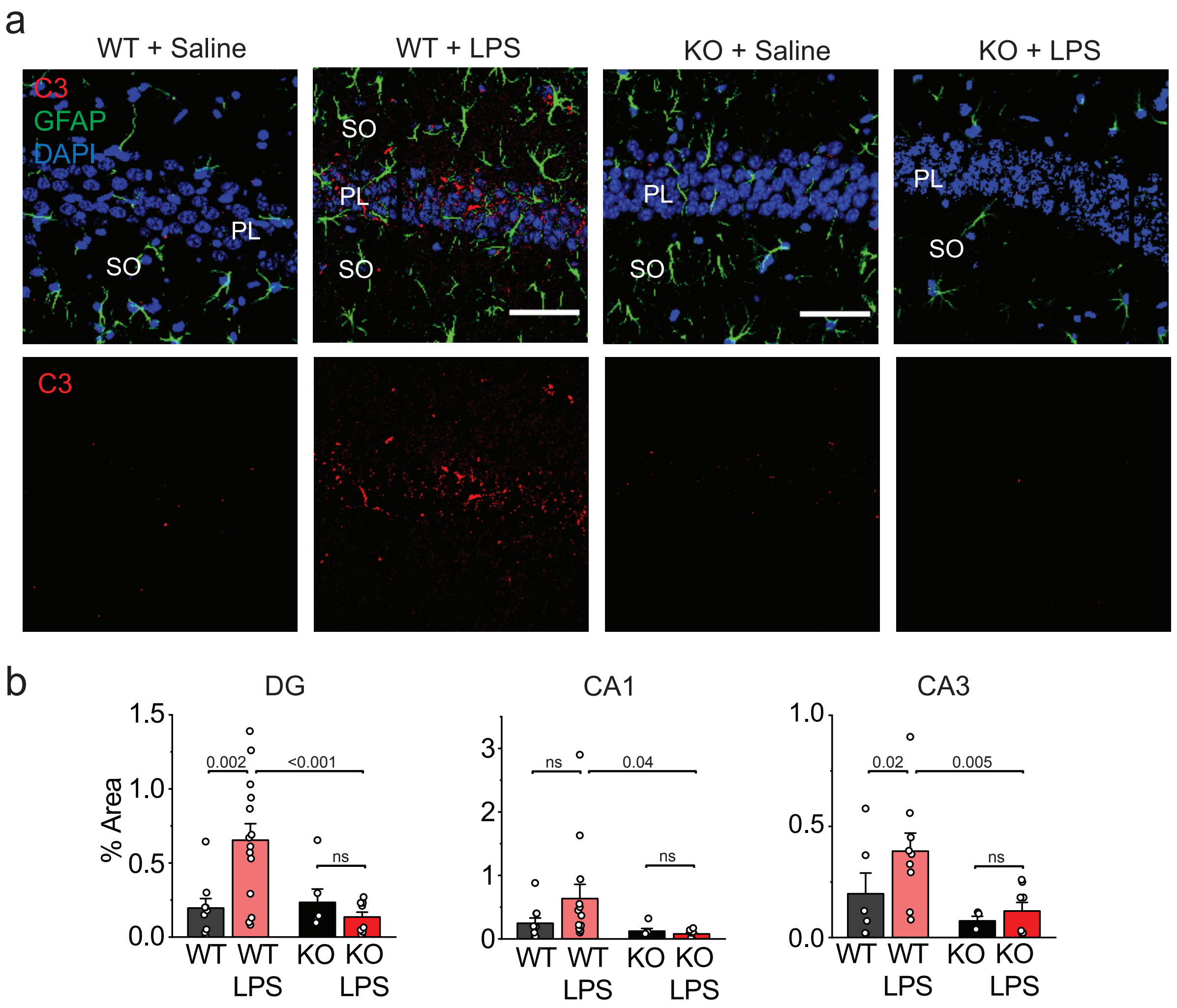

Supplementary Figure 10: Induction of complement factor C3 by LPS is blunted in male cKO mice. (a) Hippocampal brain slices from Orai1$^{fl/fl}$ (WT) and Orai1$^{fl/fl\ Aldh1l1\text{-}Cre/ERT2}$ (KO) male mice were labelled for complement C3 and GFAP. Panels show images from the CA1 hippocampus for vehicle- or LPS-treated WT and Orai1 cKO mice. The lower row shows the same images without DAPI or GFAP signals. Scale bar = 50 µM. **(b)** Quantification of C3 expression. C3 expression was quantified by measuring the fractional area of the ROI occupied by thresholded fluorescent signal. Data are given as means +/- SEM (n = 5-9 WT saline, 8-13 WT LPS, 4-6 KO saline, 8-11 KO LPS images from 3 mice/group; all data are from male mice). Statistical tests were conducted by two-way ANOVA followed by Tukey posthoc tests in each graph. Source data are provided as a Source Data file.

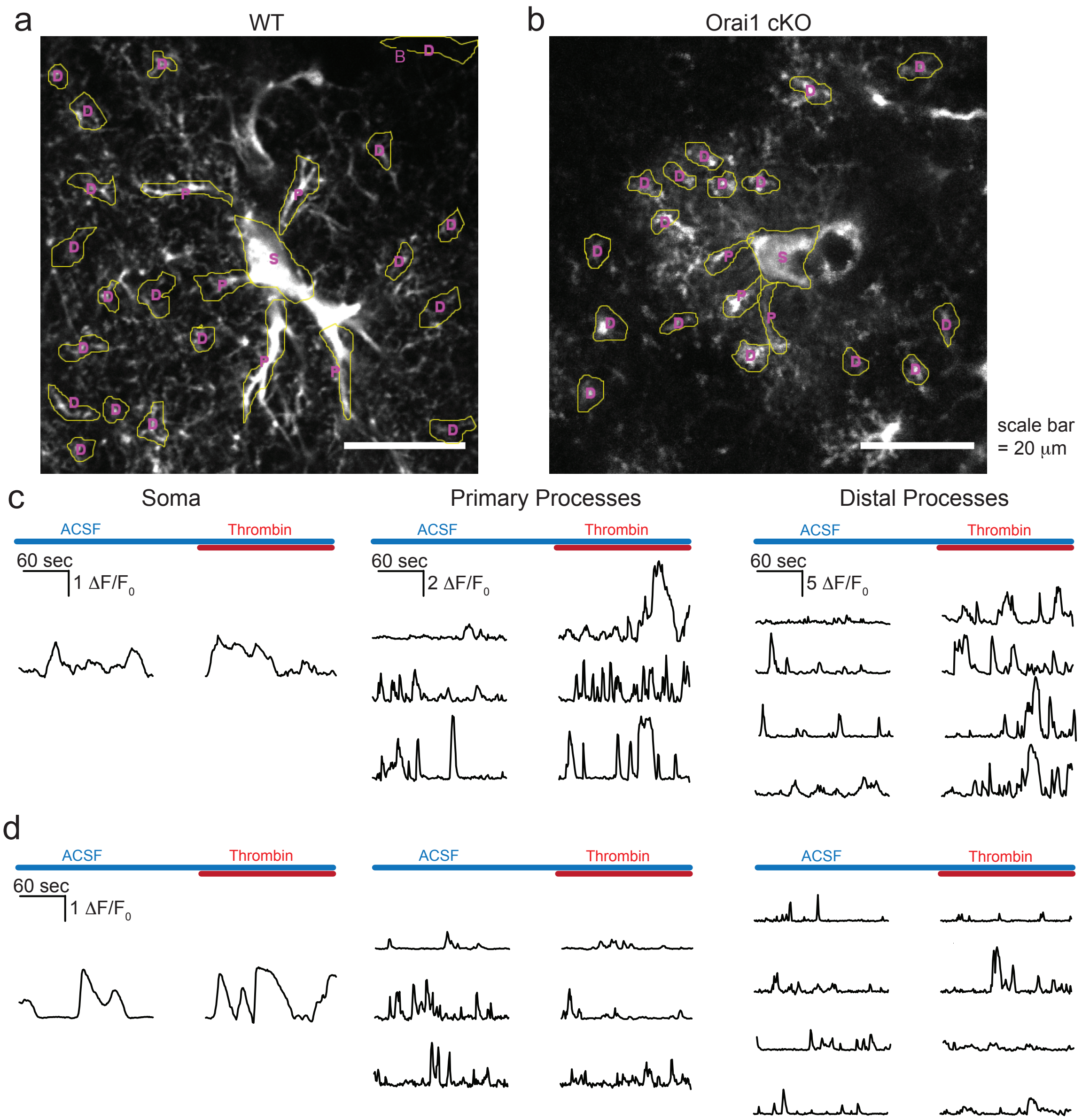

Supplementary Figure 11: Orai1 channels mediate thrombin-evoked increases in astrocyte Ca^{2+} signaling. GcaMP6f was expressed in astrocytes of the hippocampal using stereotaxic injections of AAV5 virus with an astrocyte-specific gfaABC1D promoter and Ca^{2+} signaling was assessed by 2PLSM. **(a,b)** Representative images of WT *(a)* and Orai1 cKO *(b)* images of astrocytes transfected with gCAMP6f in the CA1 region of the hippocampus illustrating regions of interest (ROIs) for analysis. ROIs (yellow outline) are drawn using a max-intensity projection image of the time series (360 sec). ROIs are labeled (in small pink text) for the soma ("S"), primary processes ("P"), and distal processes ("D"). Scale bar=20 µm. **(c,d)** Traces of the $\Delta F/F_0$ values from the soma, primary branches, and distal branches of astrocytes in brain slices from WT *(c)* and Orai1 cKO *(d)* mice. After capturing baseline activity (180 sec), acquisition was paused for ~60 sec for administration of 10 U/mL thrombin, after which acquisition was re-commenced to capture activity in the presence of thrombin (180 sec). Sample traces illustrate the previously described finding that thrombin induces increased Ca^{2+} activity in WT but not Orai1 cKO astrocytes (Ref. 23). Similar results were observed in slices from 3 mice/genotype.

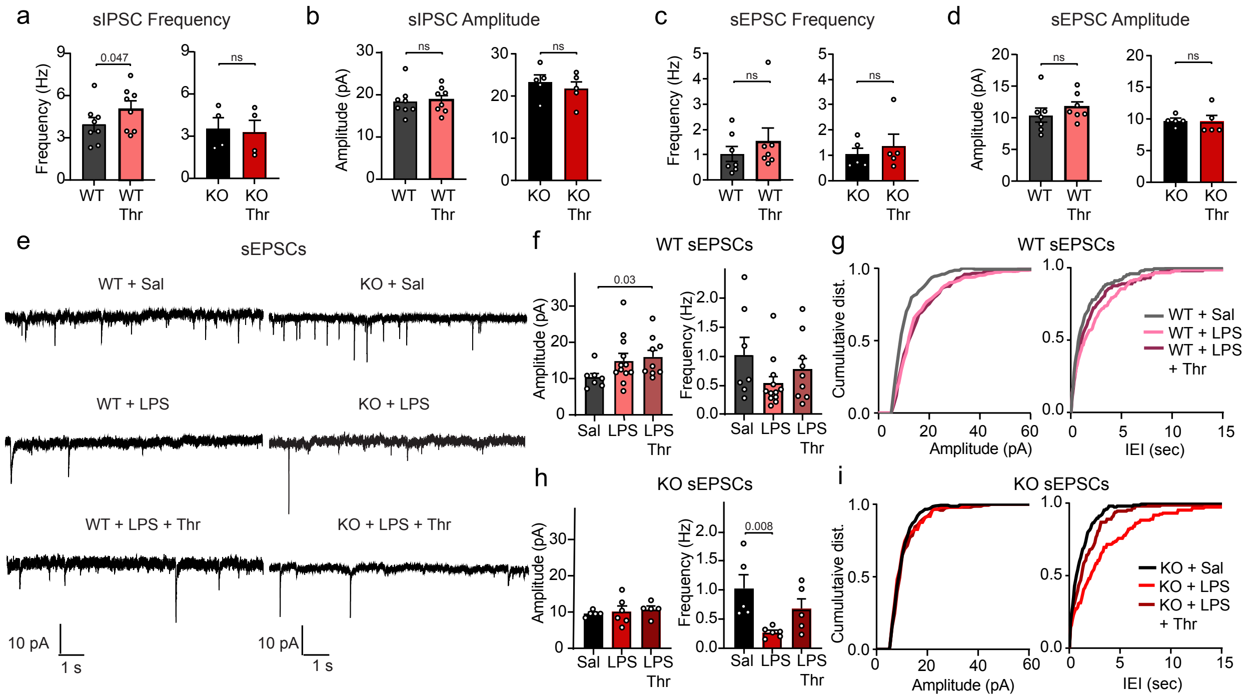

Supplementary Figure 12: Analysis of changes in excitatory and inhibitory synaptic transmission evoked by LPS or thrombin. sEPSCs and sIPSCs in hippocampal CA1 pyramidal neurons were assessed using whole cell recordings in brain slices from WT and Orai1 cKO (Orai1$^{fl/fl}$ $^{GFAP\text{-}Cre}$) mice. **(a)** In naive mice, thrombin application increases sIPSC frequency in WT, but not Orai1 cKO mice. (**b**) sIPSC amplitude, **(c)** sEPSC frequency, and **(d)** sEPSC amplitude are unchanged by thrombin. **(e-i).** Electrophysiological analysis of sEPSCs and sIPSCs in mice administered with LPS. Recordings were carried out 18 hours after LPS or saline administration. ***(e)*** Example traces of sEPSCs in the indicated conditions and genotypes. **(f-i)** Summary bar graphs *(f,h)* and cumulative distributions of the amplitude and inter-event interval of sEPSCs *(g,i)*. LPS and thrombin administration enhances the amplitude of sEPSC currents in CA1 pyramidal neurons in WT but not cKO mice. A slight trend towards smaller sEPSC frequency is seen in the Orai1 cKO groups with LPS injection which is reversed by thrombin. Data are shown as mean +/- SEM. (*a-d*) n=8 WT from 3 male & 1 female mice, 4-5 cKO cells from 4 male mice. Statistical analysis for *a-d* was done by paired two-sample t-tests. (*f-i*) n=7 WT cells from 3 male & 1 female, 12 WT+LPS cells from 2 female & 3 male mice, 9 WT+LPS+Thr cells from 2 female & 3 male mice, 5 cKO cells from 4 male mice, 6 cKO+LPS cells from 2 male & 1 female mice, 5 cKO+LPS+Thr cells from 2 male & 1 female mice. Statistical analysis of the graphs shown in *f,h* was done by one-way ANOVA followed by Tukey test. Source data are provided as a Source Data file.

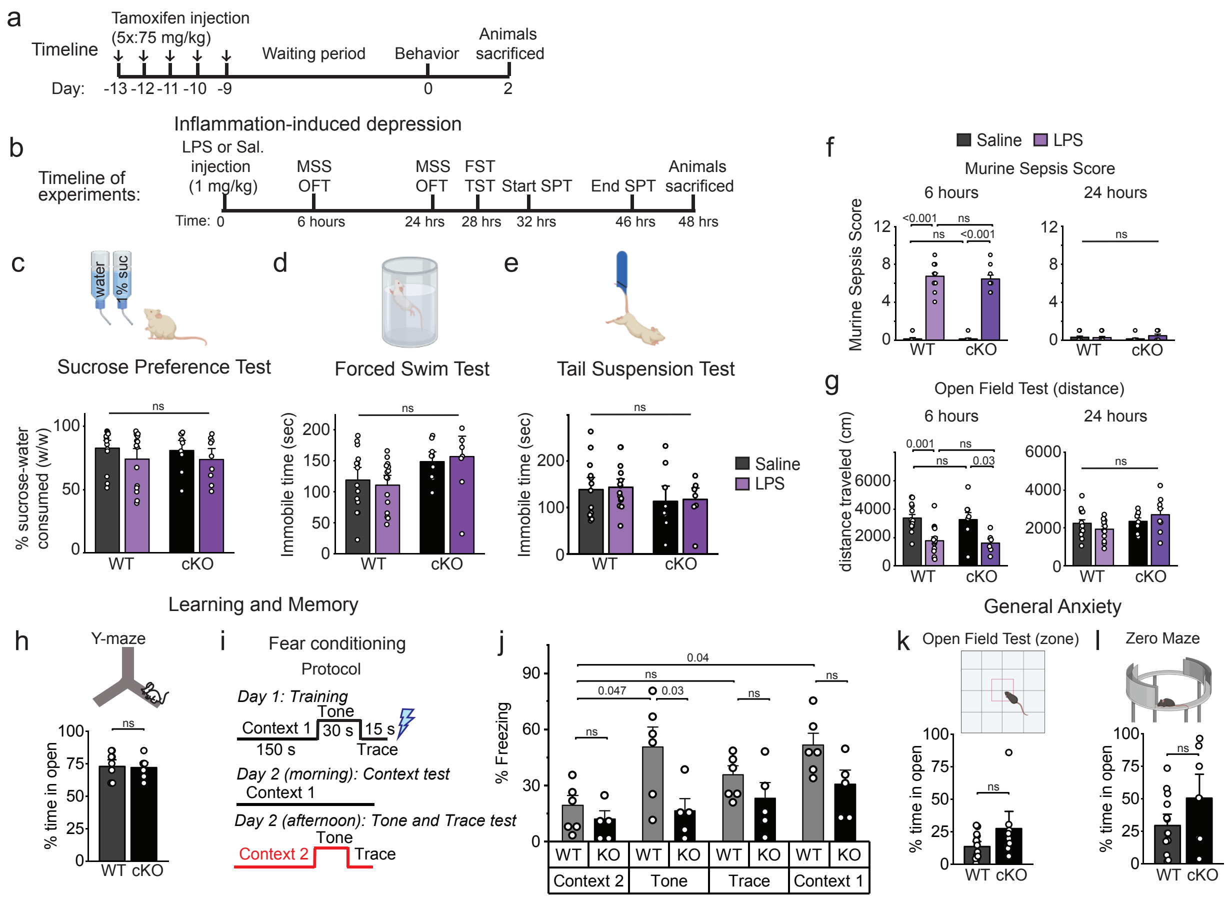

Supplementary Figure 13: Female mice do not show LPS-induced behavioral depression. (a,b) Timeline of tamoxifen injections and behavioral testing. (**c-e**) Behavioral analysis of female mice for the Sucrose Preference Test (SPT) *(c)*, Forced Swim Test (FST) *(d)*, and Tail Suspension Test (TST) *(e)*. Unlike male mice, LPS fails to evoke depression-like behaviors in female mice, consistent with previous reports (refs. 70, 71). Further, there are no differences between Orai1 cKO and WT mice. n=14 WT saline mice, n=15 WT LPS mice. n=9 Orai1 KO saline mice, n=9 Orai1 KO LPS mice. **(f)** Murine Sepsis scores (MSS) measured at 6 and 24 hours after LPS/saline injection. LPS-injected mice show elevated sickness scores at 6 but not 24 hours post-injection. No difference was seen between WT and cKO mice. **(g)** Distance traveled in the open field test is reduced in LPS-treated mice 6 hours after injection but returns to normal 24 hours after LPS administration. Further, no differences were seen in WT and Orai1 cKO mice. **(h)** Astrocyte Orai1 cKO mice do not show deficits in working memory measured by complete alterations in the Y-maze (n=9 WT, 7 Orai1 KO mice). **(i-j)** Deletion of Orai1 in astrocytes does not affect associative memory. *(i)* Protocol for fear-conditioning. N=6 WT, 5 KO mice. On day 1, mice in a chamber (context 1) were exposed to a conditioning stimulus (tone and a trace internal) followed by an unconditioned stimulus (foot shock (blue)). On day two, 24 hours after conditioning, freezing response to context 1 was assessed. Later (same day), 28-30 hours after conditioning, freezing responses to the conditioned tone and trace in a novel context 2 were assessed. *(j)* Freezing durations for probe trial (context 1) was compared to the freeze duration in context 2 prior to the tone. Orai1 cKO mice are not different from WT mice during trace or context 1 probe trials (n=6 WT, 5 cKO mice). **(k,l)** Orai1 cKO mice show no differences from WT mice in general anxiety as assessed by the time spent in the open zone in the open-field or zero-maze tests (n=14 WT, 8 cKO mice). (*c-l*). All data are given as mean +/- SEM. All data are from female mice. Statistical tests of graphs shown in *c-g* and panel *j* were conducted by two-way ANOVA followed by Tukey posthoc tests in each graph. Data in *h*,*k*, and *l* was analyzed using two-tailed unpaired t-tests. Illustrations created with BioRender.com. Source data are provided as a Source Data file.

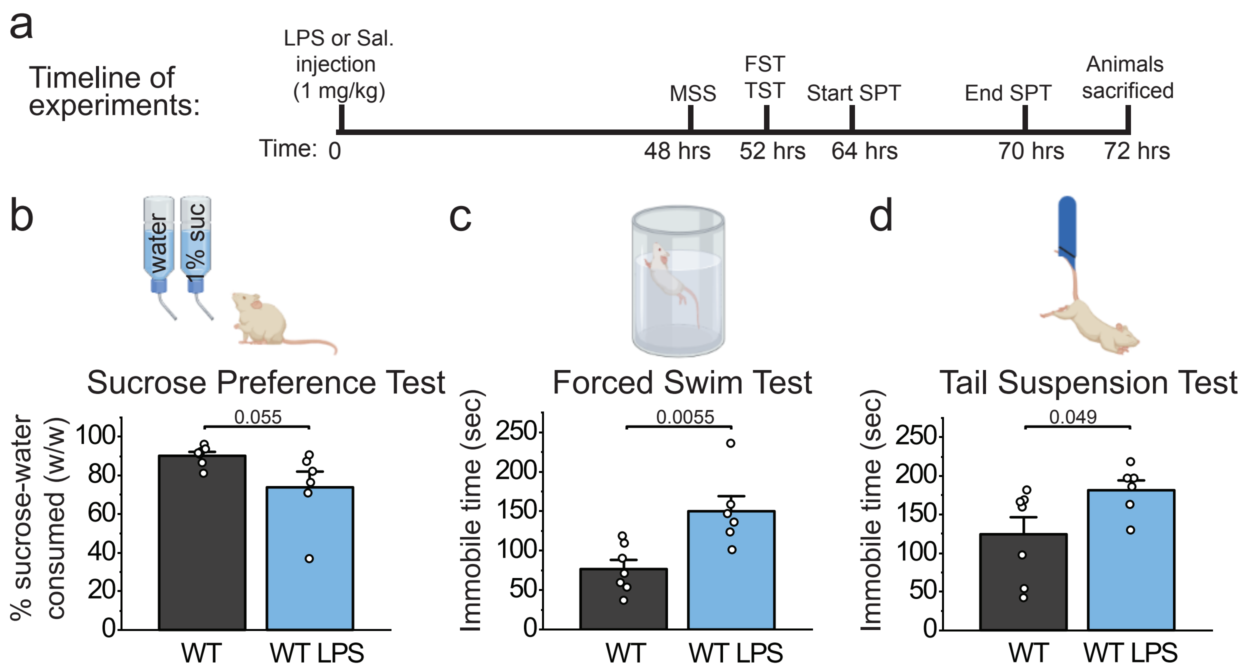

Supplementary Figure 14: Depression behaviors in male mice persist 48 hours after LPS administration in WT mice. (a) Timeline of behavioral tests after LPS injection. Mice were injected (i/p) 48 hours before commencement of behavioral testing. **(b-d)** Depression-like behaviors persist 48 hours post-injection compared to vehicle treated controls. *(b)* Anhedonia measured by sucrose preference is modestly decreased after LPS. *(c-d)* Helplessness, measured by immobile time in the forced swim *(c)* and tail suspension tests *(d)* is greater in LPS-treated mice relative to vehicle treated controls two days after LPS injection and more than one day after sickness behaviors subside. (n=7 saline treated mice, 6 LPS treated mice; all mice were male). Data are presented as mean values +/- SEM. Statistical tests were conducted by two-tailed, unpaired T-tests. Illustrations created with BioRender.com. Source data are provided as a Source Data file.

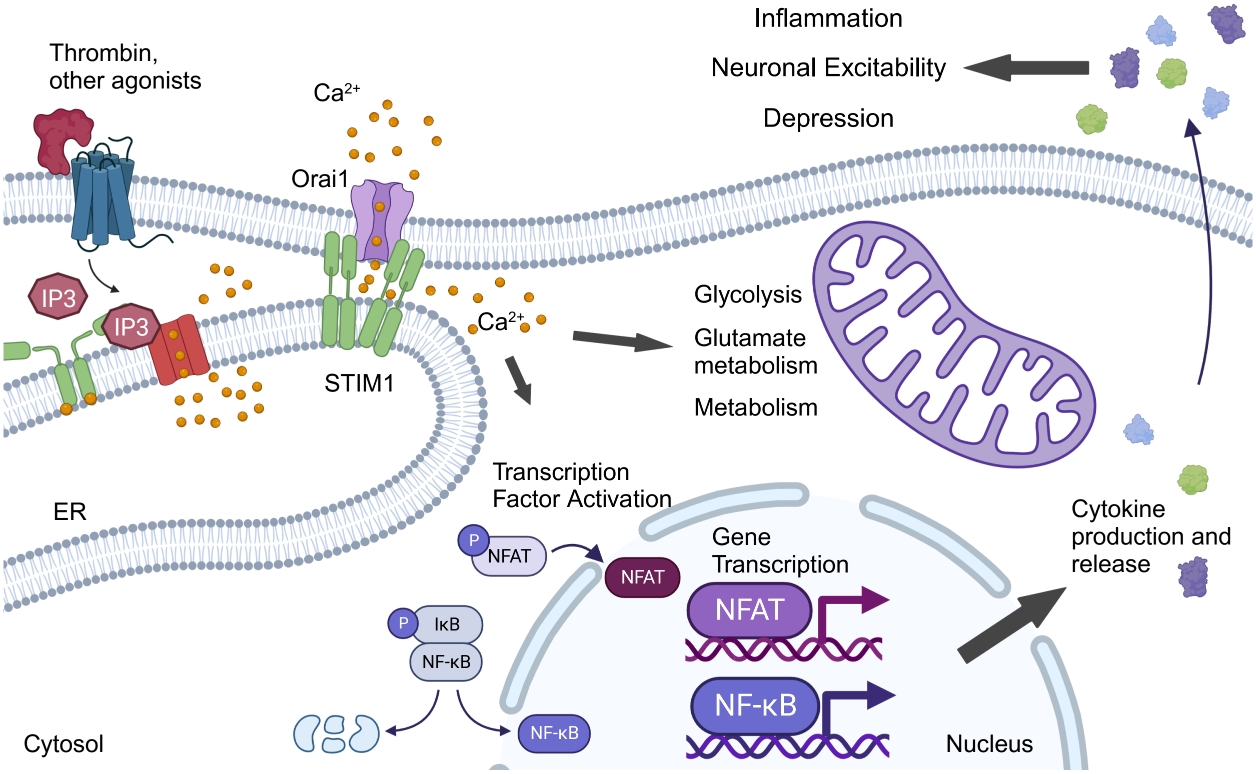

Supplementary Figure 15: A schematic summarizing the role of Orai1 for astrocyte-mediated brain inflammation. Orai1 activation is initiated by agonist stimulation of membrane receptors linked to depletion of ER Ca^{2+} stores. The ensuing Ca^{2+} signaling activates metabolic pathways and transcription factors (NFAT, NFκB) to stimulate reactive astrogliosis. These cellular cascades induce the production and release of inflammatory cytokines to increase brain inflammation and alter neuronal excitability causing behavioral depression. Illustration created with BioRender.com.

Supplementary Table 1: Selected Reactome terms enriched in WT-stimulated over Orai1 KO-stimulated astrocytes.

Reactome Pathway	R:ID	Enrichment score	Adjusted p-value
**Pathways related to inflammation and immunity**			
Antiviral mechanism by IFN stimulated genes	R-MMU-913531	-2.1274658	3.12E-06
Dectin 1 mediated noncanonical NF-κB signaling	R-MMU-5607761	-1.86233753	0.0016088
Interleukin 1 signaling	R-MMU-9020702	-1.8172633	0.00057818
P75NTR signals via NF-κB	R-MMU-193639	-1.763715067	0.026906
NF-κB is activated and signals survival	R-MMU-209560	-1.7458424	0.0287266
TGF-beta receptor signaling activates SMADS	R-MMU-2173789	-1.7739927	0.0167095
Rip mediated NF-κB activation via zbp1	R-MMU-1810476	-1.79804403	0.0191572
Interleukin 12 family signaling	R-MMU-447115	-1.790127772	0.0058957
Interleukin 12 signaling	R-MMU-9020591	-1.7890551	0.012650425
Fceri mediated NF-κB activation	R-MMU-2871837	-1.720290850	0.00522324
Signaling by TGF-beta receptor complex	R-MMU-170834	-1.57624119	0.02757275
Interleukin 1 family signaling	R-MMU-446652	-1.687015023	0.001143363
Dex H box helicases activate type I IFN and inflammatory cytokines production	R-MMU-3134963	-1.6946732	0.02927464
Signaling by interleukins	R-MMU-449147	-1.60117306	1.366E-05
Tnfr2 non-canonical NF-κB pathway	R-MMU-5668541	-1.60664659	0.00874368
Cytokine signaling in immune system	R-MMU-1280215	-1.5583488	3.535E-07
Autophagy	R-MMU-9612973	-1.488355397	0.0148089
Adaptive immune system	R-MMU-1280218	-1.4274465	0.000105492
Signaling by receptor tyrosine kinases	R-MMU-9006934	-1.399602584	0.00100610
Innate immune system	R-MMU-168249	-1.27232647	0.00474601
**Pathways related to metabolism**			
Metabolism of polyamines	R-MMU-351202	1.87817323	0.001527
Mitochondrial fatty acid beta oxidation	R-MMU-77289	1.9144498	0.001972
Fatty acid metabolism	R-MMU-8978868	1.7736444	0.000150
Diseases of metabolism	R-MMU-5668914	1.444019	0.01107
ROS and RNS production in phagocytes	R-MMU-1222556	-1.72597337	0.02001
Metabolism of amino acids and derivatives	R-MMU-71291	-1.674	9.47E-06
Selenoamino acid metabolism	R-MMU-2408522	-2.3908001	1.56E-10
Metabolism of RNA	R-MMU-8953854	-2.8574819	1.62E-47
Glycolysis	R-MMU-70171	-3.4493922	0.063
Defects in biotin metabolism	R-MMU-3323169	1.7884051	0.02036
**Pathways related to cell cycle**			
Cell cycle mitotic	R-MMU-69278	-1.2792991	0.0422387
Cell cycle	R-MMU-1640170	-1.3308853	0.004099
DNA replication	R-MMU-69306	-1.43861265	0.02872
Synthesis of DNA	R-MMU-69239	-1.4639623	0.040247
M phase	R-MMU-68886	-1.4320692	0.0028504

Separation of sister chromatids	R-MMU-2467813	-1.482707	0.011070
Cell cycle checkpoints	R-MMU-69620	-1.499805	0.005032
Mitotic metaphase and anaphase	R-MMU-2555396	-1.617434	0.00066182
DNA replication pre-initiation	R-MMU-69002	-1.6066561	0.0239563
G1 s dna damage checkpoints	R-MMU-69615	-1.6374579	0.0161582
G2 m checkpoints	R-MMU-69481	-1.646494	0.00244309
The role of gtse1 in G2-M progression after G2 checkpoint	R-MMU-8852276	-1.73928494	0.00350563
Mitotic prophase	R-MMU-68875	-1.7226644	0.00503216

Gene-set enrichment analysis. Selected pathways related to inflammation, metabolism, and cell cycle are shown. Statistical analysis was performed for all gene sets simultaneously using gene-level Wald statistics as rankings. Negative enrichment score indicates the pathway was enriched in WT-treated over KO-treated astrocytes.

Supplementary Table 2: GO pathways enriched in WT-stimulated over KO-stimulated astrocytes.

GO Pathway	GO:ID	Adjusted p-value
**Pathways related to inflammation and immunity**		
Response to cytokine	GO:0034097	1.16E-05
Cellular response to cytokine stimulus	GO:0071345	0.0001379
Regulation of type 2 immune response	GO:0002828	0.0132027
Type 2 immune response	GO:0042092	0.0155432
Type I Interferon production	GO:0032606	0.0283468
Regulation of type I interferon production	GO:0032479	0.0335079
Regulation of autophagy	GO:0010506	0.0300498
Regulation of macroautophagy	GO:0016241	0.0399612
Macroautophagy	GO:0016236	0.0001267
Autophagy	GO:0006914	0.0001304
Process utilizing autophagic mechanism	GO:0061919	0.0001304
**Pathways related to metabolism**		
Cellular metabolic process	GO:0044237	5.43E-95
Primary metabolic process	GO:0044238	3.03E-92
Organic substance metabolic process	GO:0071704	7.32E-85
Metabolic process	GO:0008152	4.79E-81
Cellular nitrogen compound metabolic process	GO:0034641	4.79E-81
Cellular protein metabolic process	GO:0044267	5.07E-76
RNA metabolic process	GO:0016070	1.32E-71
Nucleic acid metabolic process	GO:0090304	5.63E-71
Nucleobase-containing compound metabolic process	GO:0006139	2.46E-65
Heterocycle metabolic process	GO:0046483	2.46E-65
Cellular aromatic compound metabolic process	GO:0006725	5.55E-62
Organic cyclic compound metabolic process	GO:1901360	2.59E-61
Protein metabolic process	GO:0019538	1.50E-60
mRNA metabolic process	GO:0016071	2.89E-60
rRNA metabolic process	GO:0016072	2.34E-49
Peptide metabolic process	GO:0006518	4.92E-43
Cellular amide metabolic process	GO:0043603	1.83E-33
Regulation of nitrogen compound metabolic process	GO:0051171	6.23E-32
Regulation of macromolecule metabolic process	GO:0060255	4.62E-31
Regulation of cellular metabolic process	GO:0031323	1.12E-30
Regulation of cellular amide metabolic process	GO:0034248	3.88E-29
**Pathways related to cell cycle**		
Cell cycle	GO:0007049	2.59E-09
Regulation of cell cycle	GO:0051726	2.88E-07
Cell cycle phase transition	GO:0044770	2.43E-05
G1/S transition of mitotic cell cycle	GO:0000082	3.33E-05
Mitotic Cell cycle	GO:0000278	
Cell cycle G1/S phase transition	GO:0044843	0.0002742
Mitotic Cell Cycle process	GO:1903047	0.0003634
Cell Cycle Process	GO:0022402	0.0004831
Mitotic cell cycle phase transition	GO:0044772	5.39E-05
Regulation of cell cycle process	GO:0010564	0.0026733

Negative regulation of cell cycle phase transition	GO:1901988	0.0034663
Regulation of mitotic cell cycle	GO:0007346	0.0043786
Cell cycle checkpoint signaling	GO:0000075	0.0080986
Regulation of cell cycle phase transition	GO:1901987	0.0089119
Negative regulation of cell cycle	GO:0045786	0.0108684
Negative regulation of cell cycle process	GO:0010948	0.0159189
Regulation of mitotic cell cycle phase transition	GO:1901990	0.0166292
Regulation of G1/S transition of mitotic cell cycle	GO:2000045	0.0242428
Positive regulation of G1/S transition of mitotic cell cycle	GO:1900087	0.0459146

Gene ontology (GO) analysis reveals terms enriched in WT-treated over KO-treated astrocytes. Selected terms related to inflammation, metabolism, and cell cycle are shown. Statistics were performed using Fisher's Exact Test with FDR adjustment.
