# Supplementary material for: Astrocyte reactivity and inflammation-induced depression-like behaviors are regulated by Orai1 calcium channels
Source: Nat Commun. 2023 Sep 7;14:5500. doi: 10.1038/s41467-023-40968-6 (PMC10485021; doi:10.1038/s41467-023-40968-6)
Supplement: Supplementary file 7 — Reporting Summary [file 41467_2023_40968_MOESM7_ESM.pdf]

nature portfolio

Corresponding author(s): Murali Prakriya

Last updated by author(s): Aug 22, 2023

Reporting Summary

Nature Portfolio wishes to improve the reproducibility of the work that we publish. This form provides structure for consistency and transparency in reporting. For further information on Nature Portfolio policies, see our Editorial Policies and the Editorial Policy Checklist.

Statistics

For all statistical analyses, confirm that the following items are present in the figure legend, table legend, main text, or Methods section.

n/a	Confirmed	
☐	☒	The exact sample size (*n*) for each experimental group/condition, given as a discrete number and unit of measurement
☐	☒	A statement on whether measurements were taken from distinct samples or whether the same sample was measured repeatedly
☐	☒	The statistical test(s) used AND whether they are one- or two-sided<br>*Only common tests should be described solely by name; describe more complex techniques in the Methods section.*
☐	☒	A description of all covariates tested
☐	☒	A description of any assumptions or corrections, such as tests of normality and adjustment for multiple comparisons
☐	☒	A full description of the statistical parameters including central tendency (e.g. means) or other basic estimates (e.g. regression coefficient) AND variation (e.g. standard deviation) or associated estimates of uncertainty (e.g. confidence intervals)
☐	☒	For null hypothesis testing, the test statistic (e.g. *F*, *t*, *r*) with confidence intervals, effect sizes, degrees of freedom and *P* value noted<br>*Give P values as exact values whenever suitable.*
☒	☐	For Bayesian analysis, information on the choice of priors and Markov chain Monte Carlo settings
☐	☒	For hierarchical and complex designs, identification of the appropriate level for tests and full reporting of outcomes
☒	☐	Estimates of effect sizes (e.g. Cohen's *d*, Pearson's *r*), indicating how they were calculated

Our web collection on statistics for biologists contains articles on many of the points above.

Software and code

Policy information about availability of computer code

Data collection	Imaging of intracellular calcium transients: data acquisition was performed on an Olympus IX71 inverted microscope and captured with Slidebook software (Denver, CO)<br>RNA-seq: Libraries were sequenced on an Illumina NextSeq 500 instrument (Illumina Inc). FASTQ files were generated using bcl2fastq 2.19.1 (Illumina).<br>Metabolomics: acquisition was performed using Xcalibur 4.1 software.<br>RT-qPCR: acquistion was performed using Maestro 1.1 software (BioRad)<br>IHC: acquisition was performed on a Nikon A1 upright confocal microscope using Nikon Elements 5.02<br>2-photon laser microscopy: data acquisition was performed using Nikon Elements 4.60 software (Nikon).<br>Electrophysiology: data was acquired with pClamp 11.1 software (Molecular Devices).<br>Behavioral analysis: data was collected using LimeLight 5 (open field, zero maze, y maze) and FreezeFrame 5 (Fear conditioning) (Actimetrics).
Data analysis	Intracellular calcium imaging: Data was analyzed using Slidebook software and plotted with Igor Pro 6.1.<br>RNA-seq: Analysis was done using custom scripts in R ver 4.1.1 using the DESeq2 1.34.0 framework (ThermoFisher).<br>Metabolomics: analysis was performed using Tracefinder 4.1 (ThermoFisher) and metaboanalyst 5.0 (metaboanalyst.ca)<br>RT-qPCR: analysis was performed using Maestro 1.1 software (BioRad)<br>IHC: images were analyzed using Nikon Elements 5.02 and ImageJ 1.53<br>2-photon laser microscopy: analysis was performed on Nikon Elements 4.60 software and OriginPro 2022 9.9.0.225.<br>Electrophysiology: analysis was performed using pClamp 11.1 and MiniAnalysis 6.0.7 (Synaptosoft)<br>Behavioral analysis: was performed using Limelight 5 (open field, zero maze) and FreezeFrame 5 (fear conditioning). Other behaviors were

scored manually.

Metabolomics graphs were generated in Matlab 2023A and further edited in Adobe Illustrator. Summary graphs were generated in OriginPro and further edited in Adobe Illustrator. Statistical analyses were performed using OriginPro 2022 9.9.0.225.

All original code has been deposited at https://github.com/PrakriyaLab/Astrocyte2022. Custom scripts for differential expression analysis can be found at https://github.com/NUPulmonary/utils. Any additional information required to reanalyze the data reported in this paper is available from the lead contact upon request.

For manuscripts utilizing custom algorithms or software that are central to the research but not yet described in published literature, software must be made available to editors and reviewers. We strongly encourage code deposition in a community repository (e.g. GitHub). See the Nature Portfolio guidelines for submitting code & software for further information.

Data

Policy information about availability of data

All manuscripts must include a data availability statement. This statement should provide the following information, where applicable:

- Accession codes, unique identifiers, or web links for publicly available datasets
- A description of any restrictions on data availability
- For clinical datasets or third party data, please ensure that the statement adheres to our policy

RNA-seq data generated in this study have been deposited at https://github.com/PrakriyaLab/Astrocyte2022 and at https://www.ncbi.nlm.nih.gov/sra under accession code PRJNA933208 and are publicly available. Metabolomic data have been deposited at https://github.com/PrakriyaLab/Astrocyte2022. Source data for all summary graphs for all experiments (excel file) are provided with this paper.

Human research participants

Policy information about studies involving human research participants and Sex and Gender in Research.

Reporting on sex and gender	N/A
Population characteristics	N/A
Recruitment	N/A
Ethics oversight	N/A

Note that full information on the approval of the study protocol must also be provided in the manuscript.

Field-specific reporting

Please select the one below that is the best fit for your research. If you are not sure, read the appropriate sections before making your selection.

☒ Life sciences ☐ Behavioural & social sciences ☐ Ecological, evolutionary & environmental sciences

For a reference copy of the document with all sections, see nature.com/documents/nr-reporting-summary-flat.pdf

Life sciences study design

All studies must disclose on these points even when the disclosure is negative.

Sample size	For mouse behavior, power analysis was performed to determine sample size after a separate pilot experiment. Due to genotypes of littermate controls, more WT mice than strictly necessary were included until the minimum number of KO mice was obtained. For analysis of mice or mouse cultures by RNAseq, metabolomics, qPCR, ELISA, electrophysiology, 2PLSM, and IHC, sample size was determined based on previous experiments.
Data exclusions	No data were excluded.
Replication	All data were collected from 3+ biologically independent samples as noted in the legend of each Figure and Supplementary Figure. Calcium imaging directly or indirectly replicated experiments from our previous study, which compared WT and KO astrocyte calcium entry from 3-6 experiments/group (Toth et al, Sci Signaling, 2019). Cellular cytokine induction experiments were performed with biological replicates as indicated. Similar experiments (identical TG+PDBu treatments in WT compared to WT astrocytes treated with an Orai1 inhibitor) corroborated our findings.
Randomization	For all mouse experiments (behavior (and subsequent IHC/ELISA), electrophysiology, and 2PLSM), mice (as de-identified numbers) were randomly and approximately evenly assigned to treatmet or control groups. For cellular experiments, including RNAseq, metabolomics, and cytokine expression, each mouse was cultured in multiple well-plates which were randomly and equally assigned to treatment or control groups.

Blinding	Investigators were blinded to genotype and treatment during behavioral analysis. Other experiments were not blinded due to the non-subjective nature of these data.

Reporting for specific materials, systems and methods

We require information from authors about some types of materials, experimental systems and methods used in many studies. Here, indicate whether each material, system or method listed is relevant to your study. If you are not sure if a list item applies to your research, read the appropriate section before selecting a response.

Materials & experimental systems

n/a	Involved in the study	
	x	Antibodies
x		Eukaryotic cell lines
x		Palaeontology and archaeology
	x	Animals and other organisms
x		Clinical data
x		Dual use research of concern

Methods

n/a	Involved in the study	
x		ChIP-seq
x		Flow cytometry
x		MRI-based neuroimaging

Antibodies

Antibodies used	Mouse anti-Orai1 Abcam Cat# ab175040; RRD:AB_2877712, 1:200<br>Goat anti-mouse Alexa 488 Thermofisher Scientific Cat# A11008; RRID:AB_143165, 1:1000<br>Goat anti-rabbit Alexa 594 Thermofisher Scientific Cat# A11005; RRID:AB_2534073, 1:1000<br>Mouse anti-GFAP Thermofisher Scientific Cat #14-9892-82; RRID:AB_10598206, 1:300<br>Rabbit anti-IBA1 Thermofisher Scientific Cat #PA5-27436; RRID:AB_2544912, 1:300<br>Mouse anti-C3 Thermofisher Scientific Cat #PA5-21349; RRID:AB_11153785, 1:300<br>Rabbit GFAP antibody ThermoFisher Cat# PA1-10019; RRID:AB_1074611, 1:300
Validation	Orai1 (Abcam): Validated in previous study from our lab: https://www.ncbi.nlm.nih.gov/pubmed/33264616?dopt=Abstract<br>GFAP (Invitrogen) from the manufacturer: This Antibody was verified by Relative expression to ensure that the antibody binds to the antigen stated. The antibody has been cited over 30 times and for IHC at least 4 times, including https://pubmed.ncbi.nlm.nih.gov/32381088/ and https://pubmed.ncbi.nlm.nih.gov/32851638/<br>GFAP (Invitrogen Rabbit anti-GFAP) from the manufacturer: This Antibody was verified by Relative expression to ensure that the antibody binds to the antigen stated. This Antibody was verified by Cell treatment to ensure that the antibody binds to the antigen stated. The antibody has been cited 20 times and for IHC 12 times.<br>IBA1 (Invitrogen) from the manufacturer: This Antibody was verified by Cell treatment to ensure that the antibody binds to the antigen stated. This antibody has been cited for use in IHC at least 21 times including https://pubmed.ncbi.nlm.nih.gov/34112797/, https://pubmed.ncbi.nlm.nih.gov/34730998/, and https://pubmed.ncbi.nlm.nih.gov/32851638/<br>C3 (Invitrogen) From the manufacturer:: This Antibody was verified by Cell treatment to ensure that the antibody binds to the antigen stated. This antibody has been cited for use in IHC at least 4 times including https://pubmed.ncbi.nlm.nih.gov/32711525/ and https://pubmed.ncbi.nlm.nih.gov/35351973/

Animals and other research organisms

Policy information about studies involving animals; ARRIVE guidelines recommended for reporting animal research, and Sex and Gender in Research

Laboratory animals	C57BL/6 mice were used for all experiments, with genetic modifications as reported:<br>Orai1 fl/fl (Amgen)<br>B6N.FVB-Tg(Aldh1l1-cre/ERT2)1Khakh/J The Jackson Laboratory JAX: 031008; RRID: IMSR_JAX:031008<br>B6.Cg-Tg(Gfap-cre)73.12Mvs/J The Jackson Laboratory JAX: 012886; RRID: IMSR_JAX:012886<br>Mice for 2PLSM, Behavior, IHC, and ELISAs were 10-14 weeks old. Mice for electrophysiology were 1.5-3 weeks old.<br><br>Animals were group-housed under standard housing conditions (12:12-hour light/dark cycle with lights on at 7:00 a.m., 30-70% humidity, and temperatures of 20° to 22°C with ad libitum access to water and food), in a sterile ventilated facility.
Wild animals	No wild animals were used in this study.
Reporting on sex	Both male and female behavior was collected and reported separately. Cellular assays did not distinguish between male and female mice except for the experiment in Supplemental Figure 6 where effects were shown to be comparable between male and female mice.
Field-collected samples	No field-collected samples were used in this study.

Ethics oversight	All research protocols were approved by the Northwestern University Institutional Animal Care and Use Committee. (Protocol IS00001817)

Note that full information on the approval of the study protocol must also be provided in the manuscript.
